# Levothyroxine use and longitudinal changes in thigh muscles in at-risk participants for knee osteoarthritis: preliminary analysis from Osteoarthritis Initiative cohort

**DOI:** 10.1186/s13075-023-03012-y

**Published:** 2023-04-11

**Authors:** Bahram Mohajer, Kamyar Moradi, Ali Guermazi, Jennifer S. R. Mammen, David J. Hunter, Frank W. Roemer, Shadpour Demehri

**Affiliations:** 1 Musculoskeletal Radiology, Russell H. Morgan Department of Radiology and Radiological Science, Johns Hopkins University School of Medicine, 601 N Caroline St., JHOC 5165, Baltimore, MD 21287 USA; 2 Tehran University of Medical Sciences, School of Medicine, Tehran, Iran; 3 Department of Radiology, Chobanian & Avedisian Boston University School of Medicine, Boston, MA USA; 4 Division of Endocrinology, Diabetes, and Metabolism, Department of Medicine, Johns Hopkins University School of Medicine, Baltimore, MD USA; 5 Rheumatology Department, Royal North Shore Hospital, St Leonards, 2065 NSW Australia; 6 Sydney Musculoskeletal Health, Arabanoo Precinct, Kolling Institute, Faculty of Medicine and Health, The University of Sydney, Sydney, 2065 NSW Australia; 7 Department of Radiology, Universitätsklinikum Erlangen & Friedrich-Alexander-Universität Erlangen-Nürnberg, Erlangen, Germany

**Keywords:** Knee osteoarthritis, Levothyroxine, MRI, Muscle degeneration, Thyroid

## Abstract

**Background:**

We examined the association between levothyroxine use and longitudinal MRI biomarkers for thigh muscle mass and composition in at-risk participants for knee osteoarthritis (KOA) and their mediatory role in subsequent KOA incidence.

**Methods:**

Using the Osteoarthritis Initiative (OAI) data, we included the thighs and corresponding knees of participants at risk but without established radiographic KOA (baseline Kellgren-Lawrence grade (KL) < 2). Levothyroxine users were defined as self-reported use at all annual follow-up visits until the 4th year and were matched with levothyroxine non-users for potential confounders (KOA risk factors, comorbidities, and relevant medications covariates) using 1:2/3 propensity score (PS) matching. Using a previously developed and validated deep learning method for thigh segmentation, we assessed the association between levothyroxine use and 4-year longitudinal changes in muscle mass, including cross-sectional area (CSA) and muscle composition biomarkers including intra-MAT (within-muscle fat), contractile percentage (non-fat muscle CSA/total muscle CSA), and specific force (force per CSA). We further assessed whether levothyroxine use is associated with an 8-year risk of standard KOA radiographic (KL ≥ 2) and symptomatic incidence (incidence of radiographic KOA and pain on most of the days in the past 12 months). Finally, using a mediation analysis, we assessed whether the association between levothyroxine use and KOA incidence is mediated via muscle changes.

**Results:**

We included 1043 matched thighs/knees (266:777 levothyroxine users:non-users; average ± SD age: 61 ± 9 years, female/male: 4). Levothyroxine use was associated with decreased quadriceps CSAs (mean difference, 95%CI: − 16.06 mm^2^/year, − 26.70 to − 5.41) but not thigh muscles’ composition (e.g., intra-MAT). Levothyroxine use was also associated with an increased 8-year risk of radiographic (hazard ratio (HR), 95%CI: 1.78, 1.15–2.75) and symptomatic KOA incidence (HR, 95%CI: 1.93, 1.19–3.13). Mediation analysis showed that a decrease in quadriceps mass (i.e., CSA) partially mediated the increased risk of KOA incidence associated with levothyroxine use.

**Conclusions:**

Our exploratory analyses suggest that levothyroxine use may be associated with loss of quadriceps muscle mass, which may also partially mediate the increased risk of subsequent KOA incidence. Study interpretation should consider underlying thyroid function as a potential confounder or effect modifier. Therefore, future studies are warranted to investigate the underlying thyroid function biomarkers for longitudinal changes in the thigh muscles.

**Supplementary Information:**

The online version contains supplementary material available at 10.1186/s13075-023-03012-y.

## Background

Levothyroxine, the primary treatment for hypothyroidism, prescribed for more than 7% of Americans, is one of the most commonly used medications in the USA [1]. In a recent report using administrative claims data, among levothyroxine users with available thyroid function test results, 30% initiated the therapy for normal thyroid function and 60% for subclinical hypothyroidism [2]. Using a dual-energy X-ray analysis scan, previous work has shown an association between levothyroxine therapy and changes in body composition primarily via a decrease in muscle and lean body mass but not fat or bone mass [3]. In addition to levothyroxine use, the underlying thyroid dysfunction, as a potential confounder or effect modifier, can be associated with a wide range of muscle impairments such as myalgia, fatigability, proximal myopathy, myxoedema, or even rhabdomyolysis [4]. Changes in thigh muscle composition in the setting of levothyroxine therapy can be significant in elderly adults, who are also at risk of KOA, in which thigh muscle dysfunction is reported as an independent risk factor for KOA incidence [5]. This is important in clinical practice since thigh muscle degeneration is known as a modifiable risk factor for KOA incidence [5, 6] that can be mitigated by using specific training interventions and neuromuscular exercises [7, 8].

No prior work has investigated the association between levothyroxine use and thigh muscles’ volume and function. MRI is recognized as a valuable tool for the precise assessment of thigh muscle changes in various metabolic and non-metabolic settings [9–13]. However, studies on thyroid dysfunction-associated changes in the thigh muscles using MRI have been limited to only case reports [14, 15]. Moreover, no prior study has assessed whether thyroid dysfunction or levothyroxine use-associated changes in the thigh muscles are associated with an increased risk of KOA incidence in an at-risk population such as the Osteoarthritis Initiative (OAI) cohort.

Using a longitudinal propensity score (PS)-matched sample of participants at risk of KOA, in this exploratory study, we determined the longitudinal changes in thigh muscle size and composition and their potential association with levothyroxine use. Furthermore, we investigated whether levothyroxine use is associated with subsequent radiographic and symptomatic KOA incidence. Finally, we explored the mediatory role of these muscle biomarkers in the potential association between levothyroxine use and KOA incidence. If successfully concluded, interventions like levothyroxine dose adjustments or specific exercises aiming at the thigh muscles might be found clinically useful in modifying thigh muscle quality and subsequently in reducing the rate of KOA incidence.

## Methods

### Study participants

The data of this longitudinal observational study was obtained from the OAI cohort, which studied 4796 women and men 45–79 years of age from all ethnic groups in four clinical centers and a data coordinating center (2004–2015 clinicaltrials.gov registration code: NCT00080171). Provided with a complete explanation of the procedures and purposes of the study, all enrolled participants gave written informed consent. The protocol of the OAI cohort has been approved by the ethics review boards of all OAI collaborating centers (approval code: 10-00532) [16]. OAI study excludes participants with either inflammatory arthropathies, bilateral end-stage KOA, ambulatory aids other than a single straight cane, positive pregnancy tests, and contraindications to MRI (https://nda.nih.gov/oai/study-details). OAI participants were recruited in three cohorts of progression (cohort with KOA at baseline), incidence (cohort without KOA at baseline but exposed to risk factors), and non-exposed (cohort without KOA and its risk factors at baseline).

**Fig. 1:**
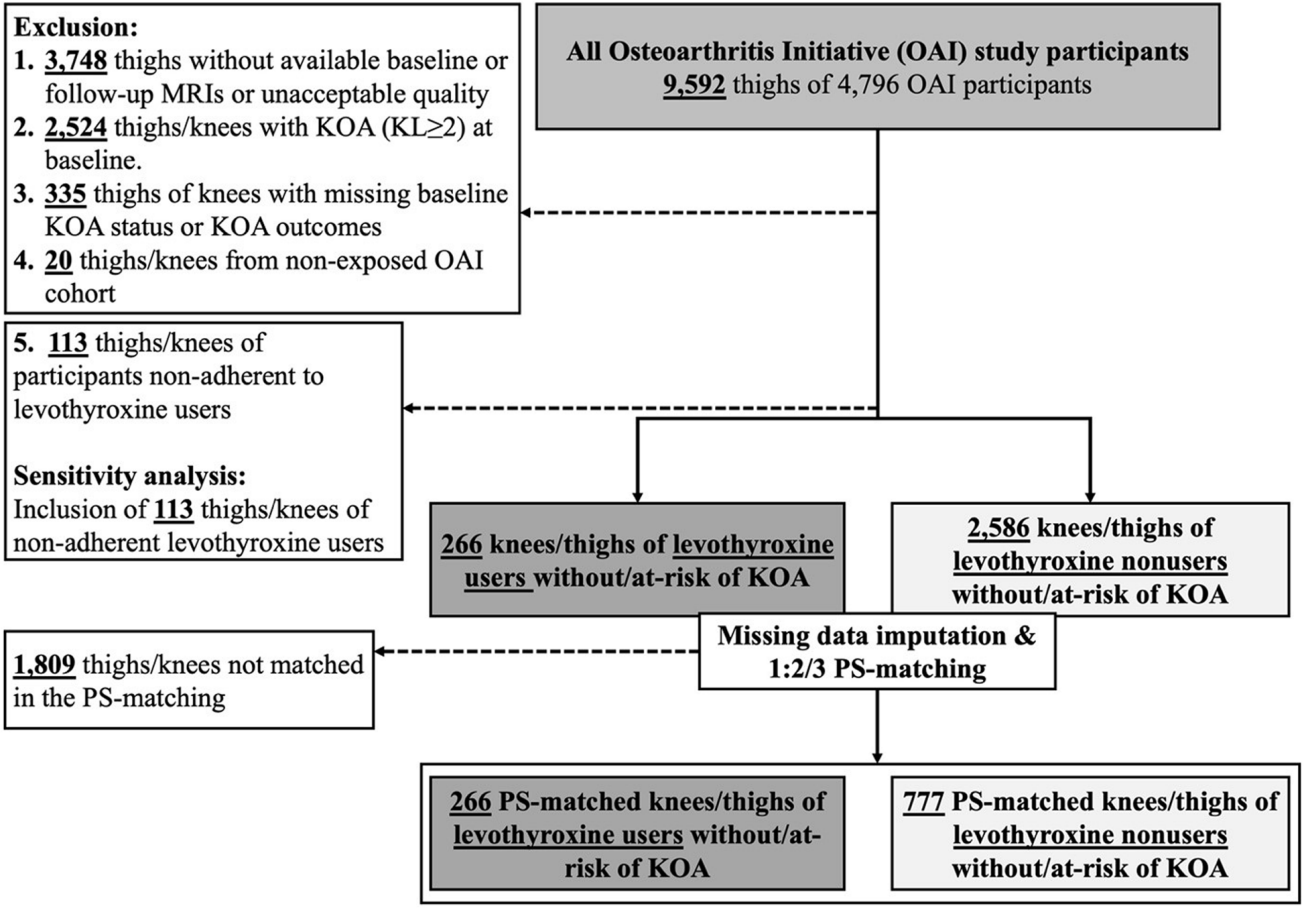
Flowchart of the study selection criteria and cohorts. KL, Kellgren-Lawrence grade; KOA, knee osteoarthritis; PS, propensity score

In this study, we further excluded participants from OAI non-exposed cohort due to the minimal risk of KOA incidence. We included the knees from the incident cohort and only the knees without radiographic KOA (semi-quantitative Kellgren-Lawrence grade (KL) < 2; the radiographs were from the knees at a fixed-flexion position) [17] in participants with unilateral OA from the progression cohort. The knees with established radiographic KOA at baseline (KL ≥ 2), unavailable KL grading, or KOA outcomes were excluded. In addition, the thighs (and corresponding knees) of participants without available baseline or 2nd- or 4th-year follow-up MRIs or with unacceptable image quality (visually assessed by two trained readers) were excluded (Fig. 1). The naming and version of the OAI dataset files used in the current study can be found in Additional file 1: Table S1.

### Exposure definition and propensity score matching

According to OAI protocol, participants brought their medications with them at baseline and annual visits. The use of levothyroxine was assessed at baseline and annual follow-up visits until year 4 of the OAI cohort, and levothyroxine use was defined as use at baseline and all annual visits while non-use was defined as no history of use during the 4-year follow-up. Non-adherent levothyroxine users in either baseline to 4th-year visits were excluded. The knees (and corresponding thighs) of levothyroxine users and non-users were matched for an exhaustive list of potential confounders, including demographic characteristics, comorbidities, risk factors, and medications (listed in Table 1 and Additional file 1: Appendix 1). After evaluating the missing data pattern in confounding variables using Little’s test [18], we implemented the multiple imputation method to estimate missing values (< 2.8% of data in each variable, Additional file 1: Table S2) since this method is associated with less biased estimates in comparison with excluding the missing data [19]. Next, the knees/thighs of participants were assigned to two matched groups of levothyroxine users and non-users, using the 1:2/3 PS-matching method on the imputed dataset (logistic regression and the nearest-neighbor matching methods). The groups were considered matched for a confounding variable as far as the standardized mean difference (SMD) between the groups for that variable was < 0.1 (Table 1).

**Table 1:** Baseline characteristics of participants before and after propensity score matching according to levothyroxine use

	OAI participants without KOA (KL < 2)					
	All participants			PS-matched participants		
	Levothyroxine non-users, ***N***: 2586	Levothyroxine users, ***N***: 266	SMD	Levothyroxine non-users, ***N***: 777	Levothyroxine users, ***N***: 266	SMD
**Demographic characteristics (included in the matching)**						
**Age (years) [mean (SD)]**	59.23 (8.99)	61.57 (8.37)	**0.269**	61.03 (8.86)	61.57 (8.37)	0.063
**No. of women [*N*** **(%)]**	1343 (51.9)	214 (80.5)	**0.632**	633 (81.5)	214 (80.5)	0.026
**Race, non-white [*N*** **(%)]**^**a**^	425 (16.4)	15 (5.6)	**0.350**	50 (6.4)	15 (5.6)	0.033
**Comorbidities and risk factors (included in the matching)**						
**PASE score [mean (SD)]**	170.96 (81.80)	154.94 (84.38)	**0.193**	158.54 (74.63)	154.94 (84.38)	0.045
**BMI (kg/m**^**2**^**) [mean (SD)]**	27.49 (4.37)	27.21 (4.10)	0.065	27.28 (4.61)	27.21 (4.10)	0.015
**Abdominal (central) obesity [*N*** **(%)]**^**b**^	1586 (61.3)	203 (76.3)	**0.328**	596 (76.7)	203 (76.3)	0.009
**Waist circumference (cm) [mean (SD)]**	99.38 (12.37)	99.57 (12.41)	0.015	99.77 (12.77)	99.57 (12.41)	0.016
**Alcohol use, ≥ 1/week [*N*** **(%)]**			0.077			0.066
< 1 drink/week	1424 (55.1)	139 (52.3)		421 (54.2)	139 (52.3)	
1–3 drinks/week	445 (17.2)	44 (16.5)		116 (14.9)	44 (16.5)	
4–7 drinks/week	379 (14.7)	43 (16.2)		134 (17.2)	43 (16.2)	
≥ 8 drinks/week	338 (13.1)	40 (15.0)		106 (13.6)	40 (15.0)	
**Smoking, current or past [*N*** **(%)]**	1148 (44.4)	112 (42.1)	0.046	313 (40.3)	112 (42.1)	0.037
**Diabetes [*N*** **(%)]**	147 (5.7)	20 (7.5)	0.074	54 (6.9)	20 (7.5)	0.022
**Malignancy [*N*** **(%)]**	82 (3.2)	4 (1.5)	**0.111**	13 (1.7)	4 (1.5)	0.014
**Charlson Comorbidity Score [mean (SD)]**	0.35 (0.84)	0.33 (0.67)	0.014	0.34 (0.73)	0.33 (0.67)	0.002
**KL grade [*N*** **(%)]**	796 (30.8)	82 (30.8)	0.001	237 (30.5)	82 (30.8)	0.007
**Knee injury [*N*** **(%)]**	602 (23.3)	48 (18.0)	**0.130**	152 (19.6)	48 (18.0)	0.039
**Medications (included in the matching)**						
**Lipid-lowering drug [*N*** **(%)]**	670 (25.9)	85 (32.0)	**0.134**	250 (32.2)	85 (32.0)	0.005
**NSAID [*N*** **(%)]**	324 (12.5)	43 (16.2)	**0.104**	127 (16.3)	43 (16.2)	0.005
**Aspirin [*N*** **(%)]**	78 (3.0)	10 (3.8)	0.041	31 (4.0)	10 (3.8)	0.012
**Systemic corticosteroid [*N*** **(%)]**	269 (10.4)	45 (16.9)	**0.191**	106 (13.6)	45 (16.9)	0.091
**Antineoplastic agents [*N*** **(%)]**	51 (2.0)	6 (2.3)	0.020	13 (1.7)	6 (2.3)	0.042
**Baseline MRI biomarkers of muscle size and composition (not included in the matching)**						
**Quadriceps CSA (mm**^**2**^**) [mean (SD)]**	5202.00 (1427.10)	4627.49 (1170.77)	**0.440**	4508.31 (1183.60)	4627.49 (1170.77)	0.099
**Quadriceps intra-MAT CSA (mm**^**2**^**) [mean (SD)]**	151.36 (111.76)	150.44 (122.11)	0.008	144.84 (104.05)	150.44 (122.11)	0.049
**Quadriceps contractile % [mean (SD)]**	96.99 (2.29)	96.69 (2.54)	**0.126**	96.73 (2.30)	96.69 (2.54)	0.018
**Flexors CSA (mm**^**2**^**) [mean (SD)]**	3253.49 (859.67)	2905.94 (724.09)	**0.437**	2871.99 (710.08)	2905.94 (724.09)	0.047
**Flexors intra-MAT CSA (mm**^**2**^**) [mean (SD)]**	143.04 (120.18)	141.58 (107.98)	0.013	142.98 (139.48)	141.58 (107.98)	0.011
**Flexors contractile % [mean (SD)]**	95.56 (3.52)	95.19 (3.17)	**0.109**	95.06 (4.47)	95.19 (3.17)	0.034
**Adductors CSA (mm**^**2**^**) [mean (SD)]**	1166.13 (599.35)	1064.90 (551.98)	**0.176**	1047.39 (530.88)	1064.90 (551.98)	0.032
**Adductors intra-MAT CSA (mm**^**2**^**) [mean (SD)]**	59.33 (45.01)	52.63 (38.50)	**0.160**	54.96 (41.78)	52.63 (38.50)	0.058
**Adductors contractile % [mean (SD)]**	94.28 (3.91)	94.36 (3.65)	0.020	94.11 (3.94)	94.36 (3.65)	0.065
**Sartorius CSA (mm**^**2**^**) [mean (SD)]**	363.72 (133.49)	306.17 (94.80)	**0.497**	306.20 (107.88)	306.17 (94.80)	0.001
**Sartorius intra-MAT CSA (mm**^**2**^**) [mean (SD)]**	27.67 (22.88)	25.31 (19.06)	**0.112**	25.47 (21.14)	25.31 (19.06)	0.008
**Sartorius contractile % [mean (SD)]**	92.38 (5.28)	91.70 (5.52)	**0.126**	91.81 (5.70)	91.70 (5.52)	0.021
**Total thigh muscles CSA (mm**^**2**^**) [mean (SD)]**	9985.34 (2693.77)	8904.50 (2198.86)	**0.440**	8733.88 (2230.31)	8904.50 (2198.86)	0.077
**intra-MAT CSA (mm**^**2**^**) [mean (SD)]**	381.40 (260.58)	369.96 (241.73)	0.046	368.25 (264.78)	369.96 (241.73)	0.007
**Total thigh muscles contractile % [mean (SD)]**	96.10 (2.54)	95.81 (2.44)	**0.116**	95.76 (2.82)	95.81 (2.44)	0.019
**Knee extension-specific contractile force (N/cm**^**2**^**) [mean (SD)]**	7.22 (2.01)	7.11 (1.87)	**0.107**	7.27 (1.95)	7.11 (1.87)	0.095
**Knee flexion-specific contractile force (N/cm**^**2**^**) [mean (SD)]**	4.73 (1.88)	4.69 (1.69)	0.021	4.62 (1.75)	4.69 (1.69)	0.042

Data are presented in numbers of thighs

A significant difference for SMD was defined as ≥ 0.1 and is shown in bold. The results show that PS-matched levothyroxine users have similar baseline characteristics in PS-matching covariates, when compared to levothyroxine non-users (SMDs < 0.1)

*BMI* body mass index, *CSA* cross-sectional area, *intra-MAT* intra-muscular adipose tissue, *JSN* joint space narrowing, *KL* Kellgren-Lawrence, *N* number of thighs, *PASE* Physical Activity Scale for the Elderly, *PS* propensity score, *SD* standard deviation, *SMD* standardized mean difference

^a^Race of participants was categorized as white and non-white considering the small number of participants in each non-white race group

^b^Abdominal obesity was defined as a waist circumference of ≥ 94 cm in men and ≥ 80 cm in women on physical examination according to the International Diabetes Foundation criteria

### Quantitative thigh muscle MRI biomarkers of size and composition

3T MRI systems (Trio, Siemens Healthcare) were used to acquire 15 continuous axial T1-weighted images from a specific region of thighs, initiating 10 cm proximal to the distal femoral epiphysis [16]. Due to the possible variation in the site of axial images between participants with different thigh lengths, an image was selected from the region of interest (ROI), which is the 33% distal length of the femur bone, based on previous studies on the OAI dataset [20, 21]. Normalization and field inhomogeneity correction was performed for the selected images using the N4ITK method [22], and a fully automated supervised deep learning algorithm was trained and validated on the images to segment all available axial thigh images in the OAI dataset [23].

Quantitative biomarkers were used to evaluate the longitudinal changes in the thigh muscles’ size and composition. MRI markers of thigh muscles’ size included the cross-sectional area (CSA) for the total and each thigh muscle group (quadriceps, flexors, adductors, and sartorius). The composition was assessed using intra-muscular adipose tissue (intra-MAT, adipose tissue inside the thigh muscles) and contractile percentage (measured by dividing the CSA of the fat-free area of the muscle by the total muscle’s CSA). Using the ImageJ software, a validated Otsu intensity thresholding method was performed on the T1-weighted images [24, 25], and the intra-MAT was calculated (in pixels) using the sum of fat-containing (white) pixels inside the muscle segments and multiplied by pixel area (mm^2^) (Additional file 1: Fig. S1). We further calculated the specific contractile force as the force generated by muscle per area unit. Participants completed isometric knee extension and flexion maximum voluntary contractions using the “Good Strength Chair” apparatus (Metitur, Jyväskylä, Finland) three times [26, 27]. The highest force of the three measurements represented the contractile force for each thigh (measured in newtons or N). The specific contractile force was calculated by dividing the maximal isometric extensor and flexor strength of the thighs by thigh muscle CSA [28]. The outcome variables are listed in Table 1.

### KOA incidence outcomes

KOA outcomes were radiographic and symptomatic KOA incidence in follow-up in participants with baseline KL grade < 2. First, we defined KOA incidence as a knee with KL grade 0 or 1 (no KOA) at baseline that developed radiographic KL grade ≥ 2 during the 8 years of the follow-up period [29]. Similar to previous studies on the OAI dataset [30, 31], symptomatic KOA incidence was defined as the incidence of both of the following (1) radiographic incidence of KOA and (2) frequent knee symptoms in the past 12 months, defined as “pain, aching, or stiffness in or around the knee on most days” for at least 1 month during the past 12 months.

### Statistical analysis

Statistical data analysis was performed using the R software version 4.0.3 (packages: *haven*, *MatchIt*, *mice*, *survival*, *lme4*, *lmerTest*, *mediation*, and *tableone*). Multilevel linear mixed-effect regression models were used to compare 4-year changes in the thigh muscle markers between levothyroxine users and non-users while considering random intercept and slope for each matched cluster of thighs of levothyroxine users: non-users and a random intercept for within-subject similarities (due to the inclusion of knees/thighs of both sides). Interaction of the time and levothyroxine use was defined as the independent predictor, while MRI biomarkers of thigh muscle size and composition were the dependent outcomes. Assumptions of regression models, including homoscedasticity, exogeneity, linearity, and normal distribution of data and residuals, were assessed and confirmed.

**Fig. 2:**
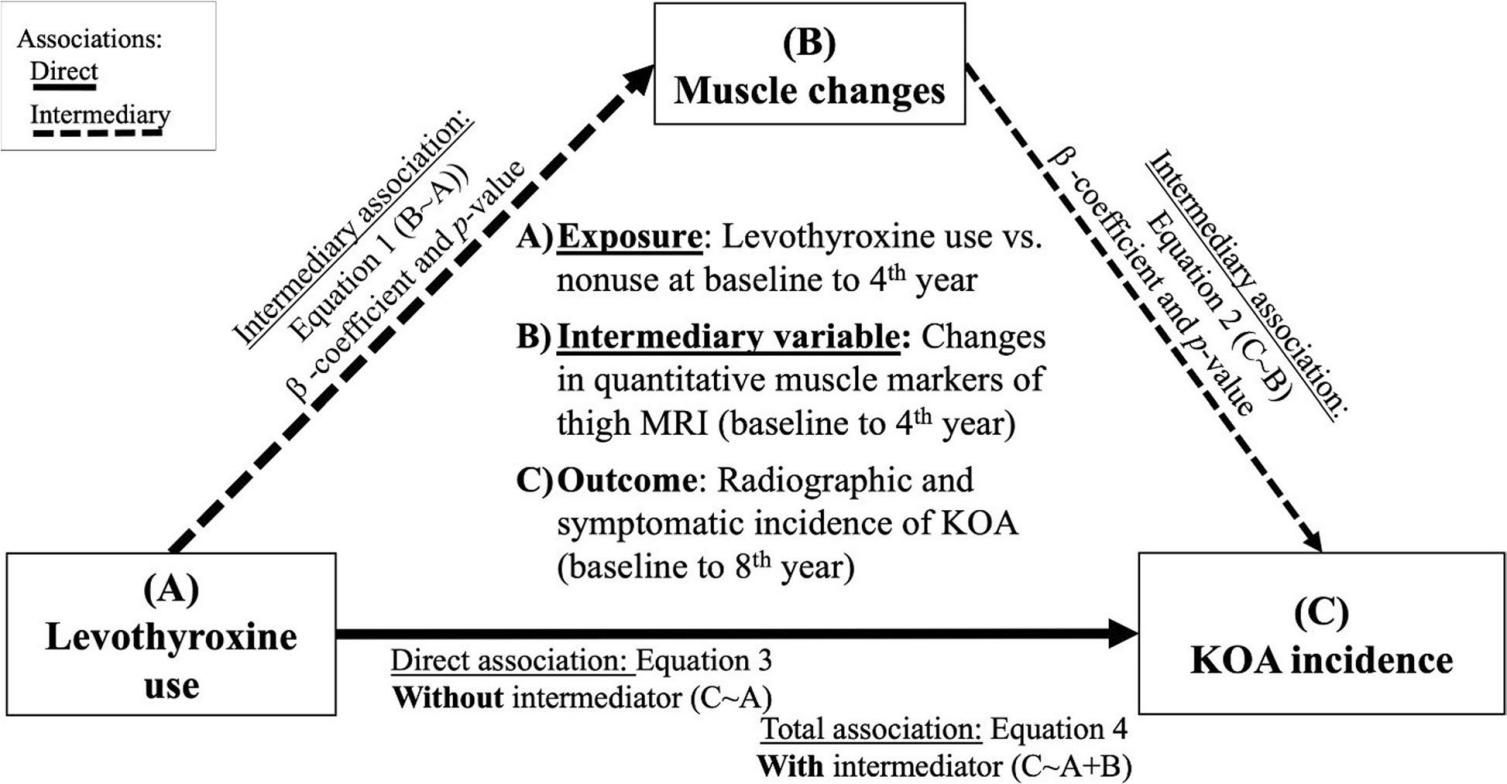
Causal mediation analysis. We evaluated the mediatory role (i.e., intermediary variable) of changes in the quantitative muscle markers of the thigh (baseline to 4th year) for the association between levothyroxine use (i.e., exposure variable) and incidence of KOA (baseline to 8th year) (i.e., outcome variable). KOA, knee osteoarthritis

KOA outcomes were assessed using survival analysis, with levothyroxine use as the independent variable and KOA radiographic and symptomatic incidence as dependent outcomes; the Cox proportional hazard model (meeting the linearity and influential observations assumptions) over 8 years was used, and the results were reported as hazard ratio (HR) and 95% confidence intervals (CI). Finally, we used mediation analysis with 1000 Monte Carlo draws for non-parametric bootstrap approximation to evaluate whether the changes in the thigh muscles have a mediatory role in the association between levothyroxine use and KOA incidence (Fig. 2).

The false discovery rate (FDR) method was used to correct *p*-values for errors related to multiple comparisons. Differences with a two-tailed FDR-corrected *p*-value below 0.05 were considered statistically significant.

To test the robustness of the results, as the sensitivity analysis, we also performed similar PS-matched analyses on all levothyroxine users, including non-adherent users from baseline to 4th-year visit (sensitivity analysis in Fig. 1).

## Results

### Baseline characteristics of participants

As depicted in Fig. 1, the knees of thighs without quality baseline or follow-up (2nd- or 4th-year) thigh MRI (*n*: 3748), with baseline KOA (KL ≥ 2, *n*: 2524), missing baseline KL grade or KOA outcomes (n:335), from non-exposed OAI cohort (*n*: 20), and/or of participants non-adherent to levothyroxine use during baseline to 4th-year visit (*n*: 113) were excluded. After 1:2/3 PS-matching, from a total of 2852 knees/thighs, 1043 were included (266:777 levothyroxine users:non-users) (Fig. 1). No statistical imbalance was found in covariates (SMDs < 0.1) following the PS matching (Table 1). More importantly, there was no significant difference in baseline biomarkers of thigh muscle MRI between the PS-matched study groups.

### The association between levothyroxine use and 4-year change in thigh muscles

Linear mixed-effect regression models showed that levothyroxine use is associated with a significant decrease in quadriceps CSA (mean difference/year, 95%CI: − 16.06 mm^2^/year, − 26.70 to − 5.41) and total thigh muscles CSA (− 22.23 mm^2^/year, − 40.25 to − 4.21, Table 2). Only the results of quadriceps CSA remained significant after FDR correction. As also shown in the table, the rest of the thigh muscle markers were not associated with levothyroxine use (FDR corrected *p* values > 0.05).

**Table 2:** Longitudinal changes in thigh muscle markers between PS-matched levothyroxine users and non-users

	Average difference/year (95%CI), ***P***			
	CSA (mm^**2**^)	intra-MAT CSA (mm^**2**^)	Contractile %	Specific strength
**Total thigh muscles**	**− 22.23 (− 40.25 to − 4.21),** ***P*: 0.016**	0.28 (− 4.16 to 4.73), *P*: 0.900	− 0.00 (− 0.05 to 0.05), *P*: 0.972	–
**Quadriceps**	**− 16.06 (− 26.70 to − 5.41),** *P*: 0.003*	1.07 (− 1.66 to 3.80), *P*: 0.443	− 0.02 (− 0.09 to 0.04), *P*: 0.438	− 0.01 (− 0.07 to 0.05), *P*: 0.709
**Flexors**	− 4.16 (− 10.95 to 2.63), *P*: 0.230	− 1.01 (− 3.49 to 1.48), *P*: 0.427	0.05 (− 0.04 to 0.13), *P*: 0.253	0.02 (− 0.03 to 0.07), *P*: 0.495
**Adductors**	− 1.74 (− 8.45 to 4.98), *P*: 0.613	0.03 (− 0.88 to 0.93), *P*: 0.952	− 0.02 (− 0.12 to 0.08), *P*: 0.703	–
**Sartorius**	− 0.25 (− 1.24 to 0.75), *P*: 0.625	0.04 (− 0.40 to 0.49), *P*: 0.857	0.00 (− 0.14 to 0.15), *P*: 0.962	–

Longitudinal mixed-effect regressions were used to assess the difference in muscle biomarkers between PS-matched levothyroxine users and non-user participants

*CI* confidence interval, *CSA* cross-sectional area, *intra-MAT* intra-muscular adipose tissue

*P values that remained significant after FDR correction, indicative of a significant difference

### The association between levothyroxine use and 8-year risk of KOA incidence

Levothyroxine use was associated with an increased risk of radiographic KOA incidence (HR, 95%CI: 1.78, 1.15–2.75). Levothyroxine use was also associated with an increased risk of symptomatic KOA incidence (1.93, 1.19–3.13) (Table 3).

**Table 3:** The association between levothyroxine use and risk of KOA radiographic and symptomatic incidence

Outcomes	Hazard ratio (95%CI), ***P***
**KOA radiographic incidence**	**1.78 (1.15–2.75),** *P*: 0.009*
**KOA symptomatic incidence**	**1.93 (1.19–3.13),** *P*: 0.007*

Cox proportional hazard model was used for analysis. Levothyroxine use, as the independent variable, was associated with an increased risk of KOA incidence over the 8-year follow-up period

*CI* confidence interval, *JSN* joint space narrowing, *KOA* knee osteoarthritis

*P values that remained significant after FDR correction, indicative of significant values

### Mediatory role of thigh muscle changes in the association between levothyroxine use and KOA incidence

Using mediation analysis on thigh muscle markers that had shown an association with levothyroxine use (i.e., quadriceps CSA and total thigh muscles CSA), we observed that 4-year changes of quadriceps CSA partially mediated the association between levothyroxine use and increased radiographic (*β* estimate, 95%CI: 0.58, 0.21–1.05) and symptomatic (0.35, 0.14–0.60) KOA incidence risk (Table 4).

**Table 4:** Mediatory role of thigh muscle MRI markers in the effect of levothyroxine use on KOA incidence

	Mediatory variables^**a**^	Estimate (95%CI), ***P***		
		Total association of levothyroxine use with KOA incidence (through Eq. 4 in Fig. **2**)	Direct association of levothyroxine use with KOA incidence^**a**^ (through Eq. 3 in Fig. **2**)	Mediatory role of thigh muscle biomarkers in the association of levothyroxine use and KOA incidence^**a**^ (through Eqs. 1 and 2 in Fig. **2**)
**KOA incidence**	**4-year changes in CSA (mm**^**2**^**)**			
**Radiographic**	**Quadriceps**	5.519 (2.751–8.082), *P* < 0.001	4.939 (2.215–7.524), *P* < 0.001	**0.580 (0.209–1.047),** ***P*** **< 0.001**
	**Total thigh muscles**	5.456 (3.136–7.837), *P* < 0.001	5.108 (2.909–7.466), *P* < 0.001	**0.348 (0.144–0.596),** ***P*** **< 0.001**
**Symptomatic**	**Quadriceps**	7.842 (3.469–11.937), *P* < 0.001	7.116 (2.915–11.306), *P* < 0.001	**0.726 (0.346–1.223),** ***P*** **< 0.001**
	**Total thigh muscles**	7.445 (4.463–11.313), *P* < 0.001	7.121 (4.119–10.977), *P* < 0.001	**0.324 (0.100–0.626),** ***P*** **< 0.001**

*CI* confidence interval, *CSA* cross-sectional area, *intra-MAT* intra-muscular adipose tissue, *JSN* joint space narrowing, *KOA* knee osteoarthritis

^a^Since mixed models could not be used in mediation analysis of the R mediate package, here, longitudinal changes in the muscle biomarkers were assessed as relative change index between baseline and 4th-year visits, i.e., (baseline − 4th-year)/baseline

### Sensitivity analysis

Sensitivity analysis indicated that the results were not sensitive to including non-adherent levothyroxine users with levothyroxine use in either baseline to 4th-year annual visits. However, the effect sizes in all analyses were comparatively smaller (Additional file 1: Table S3).

## Discussion

In this exploratory study, using a propensity score-matched design on a large sample of participants at risk of KOA, we found that levothyroxine use may be associated with a longitudinal decrease in quadriceps muscle size but not changes in their composition. In addition, participants with levothyroxine use also exhibited an increased risk of KOA incidence, partially mediated by the loss of quadriceps muscle mass, observed as a reduction in its CSA. The fact that there was no significant imbalance between MRI muscle biomarkers of levothyroxine users and non-users at baseline reduces the risk of selection bias and reinforces that the observed longitudinal changes were associated with levothyroxine use and not heterogeneity between study cohorts’ characteristics at baseline. Besides, even though the effects of potential covariates on the study results were minimized using the PS-matching method, there was a considerable heterogeneity in some of these variables such as gender and race, which necessitates further research works stratifying the participants based on these variables and studying the levothyroxine use-associated changes in each subgroup. Due to the exploratory nature of this study, the reported results cannot be interpreted as causal inferences and should be interpreted with caution as the underlying thyroid function status can play a potential role as a confounder or effect modifier for the association in this observational study.

OAI cohort provides a unique and robust database with a large sample size, long-term follow-up, a detailed list of confounders, and various demographic, clinical, and ancillary data. Given the availability of the prospectively designed optimized MRI protocol for skeletal muscle and adipose tissue segmentation [16], we developed a fully automated deep learning model of thigh segmentation to include all eligible participants in the analyses, used the PS-matching method to minimize potential confounding bias, and performed sensitivity to assess the robustness of our results.

The pathogenesis of muscle changes in patients with levothyroxine use has been only sparsely investigated [3]. Thyroid hormones are a major regulator of glucose metabolism and oxidative phosphorylation [32, 33], muscle units’ function and plasticity, and connective tissue metabolism [34], and clinical overt thyroid dysfunction has a known association with generalized myopathy. More importantly, in accordance with our findings, while loss of quadriceps muscle mass was observed, there was minimal fat infiltration and adipose changes in the muscle [35].

In addition to muscle changes, only a few case series and cross-sectional studies have investigated the association between thyroid dysfunction and KOA [36–38], and no prior study has focused on levothyroxine use and KOA outcomes. The overall results have also been inconclusive, probably due to incomplete adjustment of potential confounders, small sample size, and heterogeneity of the target population considering OA risk factors [36, 39, 40]. This longitudinal exploratory study aimed to test the hypothesis for the potential “levothyroxine use-loss of quadriceps muscle mass-KOA incidence” pathophysiological pathway. However, any causal interpretation is limited by the study design and the lack of available data on thyroid function as the potential confounder or effect modifier in the OAI database. The quadriceps muscles stabilize the knee joint during weight-bearing; therefore, its changes can be considered an independent risk factor for knee joint OA incidence [41–43]. Besides, as the mediatory role of loss of quadriceps muscle mass on KOA incidence was partial, there are other mechanisms mediating levothyroxine use and the KOA association. Previous experimental studies of KOA have shown the essential role of thyroid hormone signaling in the modulation of subchondral bone [44, 45], articular cartilage [46], and synovial fibroblasts [47]. Therefore, further exploration of several alternative mechanisms can help better delineate this suggested association pathophysiological pathway.

Using thigh MRI biomarkers of size and composition, for the first time, we showed levothyroxine use is associated with loss of quadriceps muscle mass. This finding bolds thigh MRI as a precise measurement tool for detecting early and subtle muscle changes. Even though the loss of quadriceps muscle mass was not accompanied by the changes in muscle composition, its association had a mediatory role in the increased KOA incidence risk associated with levothyroxine use. If adequately detected, the levothyroxine use-associated changes in quadriceps muscle mass may be potentially modifiable through levothyroxine dose adjustments or specific exercises and interventions aiming at the thigh muscles. Future trials are needed to assess the causal role of thigh muscle changes in this mediation.

This exploratory observational study has several limitations. First and perhaps most importantly, thyroid function assessments (e.g., serum TSH and thyroid hormone levels, thyroid iodine uptake) are not available in the OAI cohort. Therefore, we can not delineate the detailed association between underlying thyroid dysfunction and muscle degeneration in levothyroxine users. Thus, the study interpretation should consider underlying thyroid function as a potential confounder or effect modifier. This limitation needs to be addressed in future studies. Second, the OAI dataset is designed and longitudinally collected to assess the association between clinical, imaging, or laboratory-based measurements and KOA development and progression. Therefore, the OAI selection criteria are not specifically designed for subjects with thyroid dysfunction, and post hoc analyses on this dataset may be inherently susceptible to selection bias. We tried to address this possible non-random subject selection using comprehensive PS matching for potential confounders. Third, even though the physical activity scale for the elderly (PASE) is primarily designed for older adults, we also used it for participants of younger ages. This is due to the fact that numerous prior studies have confirmed the utility of the PASE in younger adults [48–51], and the OAI cohort provides the PASE scale score for all participants regardless of age. Finally, considering the absence of 3D volumetric assessments in the OAI data, we conducted a thigh MRI on a specific anatomical location at 33% distal length of the femur bone. While the exact MRI interpretation of the thigh was not feasible at other levels, previous studies have shown that muscle CSAs calculated at this level are strongly correlated with 3D muscle volume and can appropriately detect anatomical variations, cross-sectional differences, and longitudinal changes [21, 52, 53].

## Conclusions

This study showed that levothyroxine use may be associated with loss of quadriceps muscle mass which may also partially mediate the increased risk of KOA radiographic and symptomatic incidence in these participants. Further trials and prospectively designed observational studies are certainly warranted to assess the role of underlying thyroid function as a potential confounder or effect modifier on muscle changes and KOA risk. Such studies are important since levothyroxine users may benefit from clinical examinations, prescription modification, and simple preventative measures aiming at mitigating loss of muscle mass and KOA.

## Supplementary Information

**Additional file 1: Appendix 1.** Variables included in the propensity-score matching. The pattern of missing data Little’s test. **Table S1.** Osteoarthritis Initiative (OAI) datasets used in the study. **Table S2.** Percentage of missing data of the covariate included in the multiple imputations and PS-matching methods. **Table S3.1.** Baseline characteristics of participants assessed in the sensitivity analysis to inclusion of all levothyroxine users (both adherent and non-adherent users) before and after propensity score matching according to levothyroxine use. **Table S3.2.** Sensitivity to inclusion of all levothyroxine users (both adherent and non-adherent users) for longitudinal changes in thigh muscle markers between levothyroxine users and nonuser participants. **Table S3.3.** Sensitivity inclusion of all levothyroxine users (both adherent and non-adherent users) for the assessment of the association between levothyroxine use and risk of KOA incidence. **Table S3.4.** Sensitivity analysis on the inclusion of all levothyroxine users (both adherent and non-adherent users) for mediatory role of thigh muscle markers in the association between levothyroxine use and KOA incidence. **Figure S1.** Study outcome variables.

## Data Availability

The datasets used and/or analyzed during the current study are available from the corresponding author upon reasonable request.

## Abbreviations

BMI: Body mass index
CI: Confidence interval
CSA: Cross-sectional area
HR: Hazard ratio
inter-MAT: Inter-muscular adipose tissue
intra-MAT: Intra-muscular adipose tissue
JSN: Joint space narrowing
KL: Kellgren-Lawrence
KOA: Knee osteoarthritis
NASS: Non-acceptable symptomatic state
OAI: Osteoarthritis Initiative
PASE: Physical activity scale for the elderly
PS: Propensity score
SMD: Standardized mean difference
