# Supplementary material for: Levothyroxine use and longitudinal changes in thigh muscles in at-risk participants for knee osteoarthritis: preliminary analysis from Osteoarthritis Initiative cohort
Source: Arthritis Res Ther. 2023 Apr 11;25:58. doi: 10.1186/s13075-023-03012-y (PMC10088133; doi:10.1186/s13075-023-03012-y)
Supplement: Supplementary file 1 — Additional file 1: Appendix 1. Variables included in the propensity-score matching. The pattern of missing data Little’s test. Table S1. Osteoarthritis Initiative (OAI) datasets used in the study. Table S2. Percentage of missing data of the covariate included in the multiple imputations and PS-matching methods. Table S3.1. Baseline characteristics of participants assessed in the sensitivity analysis to inclusion of all levothyroxine users (both adherent and non-adherent users) before and after propensity score matching according to levothyroxine use. Table S3.2. Sensitivity to inclusion of all levothyroxine users (both adherent and non-adherent users) for longitudinal changes in thigh muscle markers between levothyroxine users and nonuser participants. Table S3.3. Sensitivity inclusion of all levothyroxine users (both adherent and non-adherent users) for the assessment of the association between levothyroxine use and risk of KOA incidence. Table S3.4. Sensitivity analysis on the inclusion of all levothyroxine users (both adherent and non-adherent users) for mediatory role of thigh muscle markers in the association between levothyroxine use and KOA incidence. Figure S1. Study outcome variables. [file 13075_2023_3012_MOESM1_ESM.docx]

# Supplementary Materials

## Appendix 1

**Variables included in the propensity-score matching**

PS-matching variables included the age (in years), gender (female/male), race/ethnicity (White or non-white race), body-mass index (BMI, Weight/(Height)^2^ in Kg/m^2^), physical activity scale for the elderly score (PASE), abdominal obesity (defined as a waist circumference of ≥94 cm in men and ≥80 cm in women according to international diabetes foundation criteria (1), yes/no), alcohol use (number of participants with ≥1/week use), current or past history of smoking (yes/no), current or past hypertension (Physical examination at OAI baseline visit, indexed as systolic blood pressure of ≥130 mm/Hg or diastolic blood pressure of ≥80 mm Hg, yes/no), diabetes (self-reported diabetes or use of anti-diabetic medications in baseline), cerebrovascular accident (self-reported history of stroke, cerebrovascular accident, blood clot or bleeding in brain, or transient ischemic attack), heart attack (self-reported history of heart attack, yes/no), heart failure (self-reported history of having heart failure or receiving treatment for heart failure, yes/no), peripheral artery disease (self-reported, yes/no), malignancy (self-reported history of cancer, other than skin cancer, leukemia or lymphoma, yes/no), chronic obstructive pulmonary disease (self-reported, having emphysema, chronic bronchitis, or chronic obstructive lung disease, yes/no), kidney dysfunction (self-reported, ever had problem with kidneys, poor kidney function based on blood high creatinine, yes/no), advanced liver dysfunction (self-reported, have cirrhosis or serious liver damage, yes/no), peptic ulcer (self-reported stomach ulcers or peptic ulcer disease, yes/no), Charlson comorbidity score (a validated self-administered questionnaire modeled on the Charlson index to evaluate the presence of comorbid conditions), KL grade (based on baseline knee X-ray, grade 0/1/2/3/4), and medications other than thyroid hormones (MIF form). Units, levels, and categories of variables are also listed in Table 1 in the main text.

**The pattern of missing data Little's test**

We assessed the pattern of missing data using a test of missing completely at random (Little's test), visual representation, and logistic regression models. The results showed a non-random pattern for missing data (2) in the OAI dataset, with fewer than 2.8% missing values for all matching variables (Supplemental Table S2). Despite the missing not at random data pattern, we included all matching variables in multiple imputation models, used according to previous studies to minimize the possible associated bias (3).

## Supplementary Tables and Figures

**Table S1.** Osteoarthritis Initiative (OAI) datasets used in the study

| **Dataset** | **Visit** | **Release version** |
| --- | --- | --- |
| All clinical | allclinical00 | 0.2.2 |
| (data regarding all clinical information and NASS incidence) | allclinical01 | 1.2.1 |
|  | allclinical03 | 3.2.1 |
|  | allclinical05 | 5.2.1 |
|  | allclinical06 | 6.2.1 |
|  | allclinical07 | 7.2.1 |
|  | allclinical08 | 8.2.2 |
|  | allclinical09 | 9.2.1 |
|  | allclinical10 | 10.2.2 |
| Medical inventory form (MIF) | MIF00 | 0.2.2 |
| (data regarding drug history) |  |  |
| Enrollees | enrollees | 25 |
| (data regarding baseline enrollment of OAI participants) |  |  |
| Knee X-ray semi-quantitative reading (Kxr sq) | Kxr sq 00 | 0.8 |
| MRI tracking and QA | mri00 | 0.2.2 |
| (data regarding availability of MRI and | mri03 | 3.2.1 |
| quality assessment) | mri06 | 6.2.1 |
| Outcomes | outcome99 | 10 |
| (data regarding all knee OA outcomes including KOA incidence) |  |  |

JSN: Joint space narrowing, NASS: Non-acceptable symptomatic state, OAI: Osteoarthritis initiative, QA: Quality assessment.

**Table S2.** Percentage of missing data of the covariate included in the multiple imputations and PS-matching methods

| **Variables** | **Missing %** |
| --- | --- |
| **Subject characteristics** |  |
| **Age** | 0.00% |
| **No. of women** | 0.00% |
| **Race, non-white** | 0.07% |
| **Comorbidities and Risk factors** |  |
| **PASE score** | 0.55% |
| **BMI** | 0.10% |
| **Abdominal (central) obesity** | 0.21% |
| **Weist circumference** | 0.21% |
| **Alcohol use, ≥1/week** | 1.16% |
| **Smoking, current or past** | 0.58% |
| **Diabetes** | 2.12% |
| **Malignancy** | 1.61% |
| **Charlson Comorbidity score** | 1.02% |
| **KL grade** | 0.00% |
| **Medication data** | 0.00% |

BMI: Body Mass Index, COPD: Chronic Obstructive Pulmonary Disease, CVA: Cerebrovascular Accident, KL: Kellgren-Lawrence grade, PASE: Physical Activity for Elderly Scale, PS: Propensity-score.

**Table S3.1.** Baseline characteristics of participants assessed in the sensitivity analysis to inclusion of all levothyroxine users (both adherent and non-adherent usres) before and after propensity score matching according to levothyroxine use.

|  | **OAI participants without KOA (KL<2)** | | | | | | | | | | | |  |
| --- | --- | --- | --- | --- | --- | --- | --- | --- | --- | --- | --- | --- | --- |
|  | **All participants** | | | | |  |  | **PS-matched participants** | | | | | |
|  | **Levothyroxine non-users** | | | | **All Levothyroxine users*** |  |  | **Levothyroxine non-users** | **All Levothyroxine users*** | | |  | |
|  | N: 2586 | | | | N: 379 | **SMD** |  | N: 991 | N: 357 | | | **SMD** | |
| **Demographic characteristics (included in the matching)** | | | |  | |  |  |  |  | | |  | |
| **Age (year) [mean (SD)]** | 59.23 (8.99) | | | | 62.09 (8.56) | **0.326** |  | 60.88 (8.94) | 61.34 (8.19) | | | 0.054 | |
| **No. of women [N (%)]** | 1343 (51.9) | | | | 296 (78.1) | **0.571** |  | 747 (75.4) | 274 (76.8) | | | 0.032 | |
| **Race, non-white [N (%)] †** | 425 (16.4) | | | | 28 (7.4) | **0.282** |  | 89 (9.0) | 28 (7.8) | | | 0.041 | |
| **Comorbidities and risk factors (included in the matching)** |  | | | |  |  |  |  |  | | |  | |
| **PASE score [mean (SD)]** | 170.96 (81.80) | | | | 156.51 (85.19) | **0.173** |  | 160.39 (75.34) | 159.22 (85.87) | | | 0.014 | |
| **BMI (kg/m^2^) [mean (SD)]** | 27.49 (4.37) | | | | 27.01 (4.27) | **0.111** |  | 27.05 (4.51) | 27.12 (4.23) | | | 0.015 | |
| **Abdominal (central) obesity [N (%)]** § | 1586 (61.3) | | | | 281 (74.1) | **0.277** |  | 712 (71.8) | 260 (72.8) | | | 0.022 | |
| **Weist circumference (cm) [mean (SD)]** | 99.38 (12.37) | | | | 99.17 (12.74) | 0.016 |  | 98.95 (13.15) | 99.17 (12.94) | | | 0.017 | |
| **Alcohol use, ≥1/week [N (%)]** |  | | | |  | 0.086 |  |  |  | | | 0.065 | |
| <1 drink/wk | 1424 (55.1) | | | | 202 (53.3) |  |  | 536 (54.1) | 196 (54.9) | | |  | |
| 1-3 drinks/wk | 445 (17.2) | | | | 58 (15.3) |  |  | 141 (14.2) | 57 (16.0) | | |  | |
| 4-7 drinks/wk | 379 (14.7) | | | | 63 (16.6) |  |  | 166 (16.8) | 55 (15.4) | | |  | |
| ≥8 drinks/wk | 338 (13.1) | | | | 56 (14.8) |  |  | 148 (14.9) | 49 (13.7) | | |  | |
| **Smoking, current or past [N (%)]** | 1148 (44.4) | | | | 159 (42.0) | 0.049 |  | 457 (46.1) | 150 (42.0) | | | 0.083 | |
| **Diabetes [N (%)]** | 147 (5.7) | | | | 25 (6.6) | 0.038 |  | 58 (5.9) | 23 (6.4) | | | 0.025 | |
| **Malignancy [N (%)]** | 82 (3.2) | | | | 9 (2.4) | 0.049 |  | 28 (2.8) | 9 (2.5) | | | 0.019 | |
| **Charlson Comorbidity score [mean (SD)]** | 0.35 (0.84) | | | | 0.34 (0.69) | 0.006 |  | 0.37 (0.88) | 0.34 (0.70) | | | 0.042 | |
| **KL grade, grade 1 [N (%)]** | 796 (30.8) | | | | 109 (28.8) | 0.044 |  | 291 (29.4) | 107 (30.0) | | | 0.013 | |
| **Knee injury [N (%)]** | 602 (23.3) | | | | 75 (19.8) | 0.085 |  | 197 (19.9) | 73 (20.4) | | | 0.014 | |
| **Medications (included in the matching)** |  | | | |  |  |  |  |  | | |  | |
| **Lipid-lowering drug [N (%)]** | 670 (25.9) | | | | 114 (30.1) | 0.093 |  | 275 (27.7) | 106 (29.7) | | | 0.043 | |
| **NSAID [N (%)]** | 324 (12.5) | | | | 67 (17.7) | **0.144** |  | 149 (15.0) | 63 (17.6) | | | 0.071 | |
| **Aspirin [N (%)]** | 78 (3.0) | | | | 12 (3.2) | 0.009 |  | 35 (3.5) | 12 (3.4) | | | 0.009 | |
| **Systemic corticosteroid [N (%)]** | 269 (10.4) | | | | 59 (15.6) | **0.154** |  | 128 (12.9) | 50 (14.0) | | | 0.032 | |
| **Antineoplastic agents [N (%)]** | 51 (2.0) | | | | 8 (2.1) | 0.010 |  | 14 (1.4) | 8 (2.2) | | | 0.062 | |
| **Baseline MRI biomarkers of muscle size and composition (NOT included in the matching)** | | | | | | |  |  |  | |  | | |
| **Quadriceps CSA (mm2) [mean (SD)]** | | 5202.00 (1427.10) | 4633.35 (1219.07) | | | **0.428** |  | 4676.12 (1194.57) | | 4702.26 (1208.11) | | 0.022 |  |
| **Quadriceps Intra-MAT CSA (mm2) [mean (SD)]** | | 151.36 (111.76) | 149.79 (115.86) | | | 0.014 |  | 144.92 (114.58) | | 152.05 (117.94) | | 0.061 |  |
| **Quadriceps contractile % [mean (SD)]** | | 96.99 (2.29) | 96.73 (2.36) | | | **0.113** |  | 96.85 (2.38) | | 96.73 (2.38) | | 0.049 |  |
| **Flexors CSA (mm2) [mean (SD)]** | | 3253.49 (859.67) | 2923.55 (762.86) | | | **0.406** |  | 2954.56 (734.48) | | 2957.02 (768.73) | | 0.003 |  |
| **Flexors Intra-MAT CSA (mm2) [mean (SD)]** | | 143.04 (120.18) | 139.47 (100.40) | | | 0.032 |  | 138.99 (140.16) | | 139.70 (102.20) | | 0.006 |  |
| **Flexors contractile % [mean (SD)]** | | 95.56 (3.52) | 95.28 (2.95) | | | **0.086** |  | 95.32 (4.23) | | 95.34 (2.96) | | 0.006 |  |
| **Adductors CSA (mm2) [mean (SD)]** | | 1166.13 (599.35) | 1081.59 (554.88) | | | **0.146** |  | 1103.98 (534.19) | | 1091.69 (561.22) | | 0.022 |  |
| **Adductors Intra-MAT CSA (mm2) [mean (SD)]** | | 59.33 (45.01) | 55.94 (41.09) | | | 0.079 |  | 56.49 (44.63) | | 56.15 (41.91) | | 0.008 |  |
| **Adductors contractile % [mean (SD)]** | | 94.28 (3.91) | 94.25 (3.60) | | | 0.007 |  | 94.27 (4.05) | | 94.30 (3.63) | | 0.008 |  |
| **Sartorius CSA (mm2) [mean (SD)]** | | 363.72 (133.49) | 309.94 (103.61) | | | **0.450** |  | 313.43 (108.00) | | 316.70 (102.21) | | 0.031 |  |
| **Sartorius Intra-MAT CSA (mm2) [mean (SD)]** | | 27.67 (22.88) | 26.94 (22.99) | | | 0.032 |  | 25.33 (20.82) | | 27.21 (23.50) | | 0.085 |  |
| **Sartorius contractile % [mean (SD)]** | | 92.38 (5.28) | 91.40 (6.00) | | | **0.173** |  | 92.09 (5.35) | | 91.59 (5.94) | | 0.088 |  |
| **Total thigh muscles CSA (mm2) [mean (SD)]** | | 9985.34 (2693.77) | 8948.43 (2327.61) | | | **0.412** |  | 9048.09 (2295.06) | | 9067.66 (2324.33) | | 0.008 |  |
| **Intra-MAT CSA (mm2) [mean (SD)]** | | 381.40 (260.58) | 372.14 (233.94) | | | 0.037 |  | 365.72 (282.30) | | 375.11 (239.33) | | 0.036 |  |
| **Total thigh muscles contractile % [mean (SD)]** | | 96.10 (2.54) | 95.83 (2.28) | | | **0.112** |  | 95.93 (2.82) | | 95.86 (2.32) | | 0.025 |  |
| **Knee extension specific contractile force (N/cm2) [mean (SD)]** | | 7.22 (2.01) | 7.01 (1.84) | | | **0.107** |  | 7.21 (2.06) | | 6.98 (1.86) | | **0.116** |  |
| **Knee flexion specific contractile force (N/cm2) [mean (SD)]** | | 4.73 (1.88) | 4.72 (1.71) | | | 0.004 |  | 4.58 (1.85) | | 4.77 (1.72) | | **0.106** |  |

Data are presented in numbers of thighs. BMI: Body-mass index, CSA: Cross-sectional area, Intra-MAT: Intra-Muscular Adipose Tissue, JSN: Joint Space Narrowing, KL: Kellgren-Lawrence, N: Number of thighs, PASE: physical activity scale for the elderly, PS: Propensity score, SD: Standard deviation, SMD: Standardized mean difference.

A significant difference for SMD was defined as ≥ 0.1 and is shown as bold. The results show that PS-matched levothyroxine users have similar baseline characteristics in PS-matching covariates, when compared to levothyroxine non-users (SMDs <0.1).

* Levothyroxine use was defined as levothyroxine use in either of baseline to 4^th^-year visits.

**†** Race of participants was categorized as white and non-white considering the small number of participants in each non-white race group.

**§** Abdominal obesity was defined as a waist circumference of ≥94 cm in men and ≥80 cm in women on physical examination according to international diabetes foundation criteria

**Table S3.2.** Sensitivity to inclusion of all levothyroxine users (both adherent and non-adherent usres) for longitudinal changes in thigh muscle markers between levothyroxine users and nonuser participants

|  |  | **Average Difference/year (95% CI), P** | | |
| --- | --- | --- | --- | --- |
|  | **CSA (mm2)** | **Intra-MAT CSA (mm2)** | **Contractile %** | **Specific strength** |
| **Total thigh muscles** | **-21.32 (-39.31 – -3.33), P:0.020** | 1.42 (-2.58 – 5.41), P:0.487 | -0.01 (-0.06 – 0.03), P:0.537 | – |
| **Quadriceps** | **-14.30 (-24.42 – -4.18), P:0.006*** | 1.74 (-0.73 – 4.22), P:0.167 | -0.04 (-0.09 – 0.02), P:0.219 | -0.00 (-0.06 – 0.05), P:0.873 |
| **Flexors** | -3.48 (-9.86 – 2.90), P:0.285 | 0.11 (-2.07 – 2.29), P:0.920 | 0.01 (-0.06 – 0.08), P:0.786 | -0.02 (-0.06 – 0.03), P:0.459 |
| **Adductors** | -2.62 (-9.30 – 4.07), P:0.443 | -0.11 (-0.98 – 0.76), P:0.810 | -0.03 (-0.12 – 0.06), P:0.514 | – |
| **Sartorius** | -0.36 (-1.38 – 0.66), P:0.489 | -0.21 (-0.63 – 0.21), P:0.321 | 0.04 (-0.09 – 0.17), P:0.508 | – |

Longitudinal mixed-effect regressions were used to assess difference in muscle biomarkers between all levothyroxine users and nonuser participants. CI: Confidence interval, CSA: Cross-sectional Area, Intra-MAT: Intra-muscular Adipose Tissue.

Levothyroxine use in either of baseline to 4^th^-year visits was the independent variable (i.e., predictor). All variables included in the PS-matching model were included as the covariate of adjustment.

***** P values that remained significant after FDR correction, indicative of a significant difference.

**Table S3.3.** Sensitivity inclusion of all levothyroxine users (both adherent and non-adherent usres) for the assessment of the association between levothyroxine use and risk of KOA incidence

| **Outcomes** | **Hazard ratio (95%CI), P** |
| --- | --- |
| **KOA radiographic Incidence** | **1.60 (1.13-2.28), P:0.008*** |
| **KOA symptomatic Incidence** | **1.66 (1.13-2.44), P:0.009*** |

CI: Confidence interval, JSN: Joint Space Narrowing, Knee osteoarthritis. Adjusted analysis were performed on all eligible knees (391 knees of levothyroxine users: 2635 knees of levothyroxine non-users) were included. All variables included in the PS-matching model were included as the covariate of adjustment. Levothyroxine use in either of baseline to 4^th^-year visits, as the independent variable, was associated with an increased risk of KOA incidence over the 8-year follow-up period, which is similar to the main results.

***** P values that remained significant after FDR correction, indicative of a significant values.

**Table S3.4.** Sensitivity analysis on the inclusion of all levothyroxine users (both adherent and non-adherent usres) for mediatory role of thigh muscle markers in the association between levothyroxine use and KOA incidence

|  |  | **Estimate (95% CI), P** | | |
| --- | --- | --- | --- | --- |
|  | **Mediatory variables†** | **Total association of levothyroxine use with KOA incidence**  (Through Equation 1 in Figure 2) | **Direct association of levothyroxine use with KOA incidence**  (Through Equation 2 in Figure 2) | **Mediatory role of thigh muscle biomarkers in the association of levothyroxine use and KOA incidence**  (Through Equasions 3a and 3b in Figure 2) |
| **KOA incidence** | **4-year changes in CSA (mm2)** |  |  |  |
| **Radiographic** | **Quadriceps** | 3.994 (2.370 – 5.884), P<0.001 | 3.832 (2.251 – 5.734), P<0.001 | **0.161 (0.023 – 0.345), P:0.020** |
|  | **Total thigh muscles** | 4.224 (2.452 – 6.274), P<0.001 | 4.310 (2.527 – 6.387), P<0.001 | -0.086 (-0.249 – 0.044), P:0.200 |
| **Symptomatic** | **Quadriceps** | 4.628 (2.466 – 7.252), P<0.001 | 4.551 (2.312 – 7.232), P<0.001 | 0.077 (-0.073 – 0.277), P:0.240 |
|  | **Total thigh muscles** | 4.742 (2.516 – 7.199), P<0.001 | 4.927 (2.571 – 7.509), P<0.001 | -0.185 (-0.410 – 0.037), P:0.100 |

Levothyroxine user were considered as participants with levothyroxine use in either of baseline to 4^th^-year visits.

CI: Confidence interval, CSA: Cross-sectional Area, Intra-MAT: Intra-muscular Adipose Tissue, JSN: Joint space narrowing, KOA: Knee osteoarthritis.

**†** Thigh muscle markers that their changes were associated with levothyroxine use selected for causal mediation analysis.

**Figure S1**. Study outcome variables.


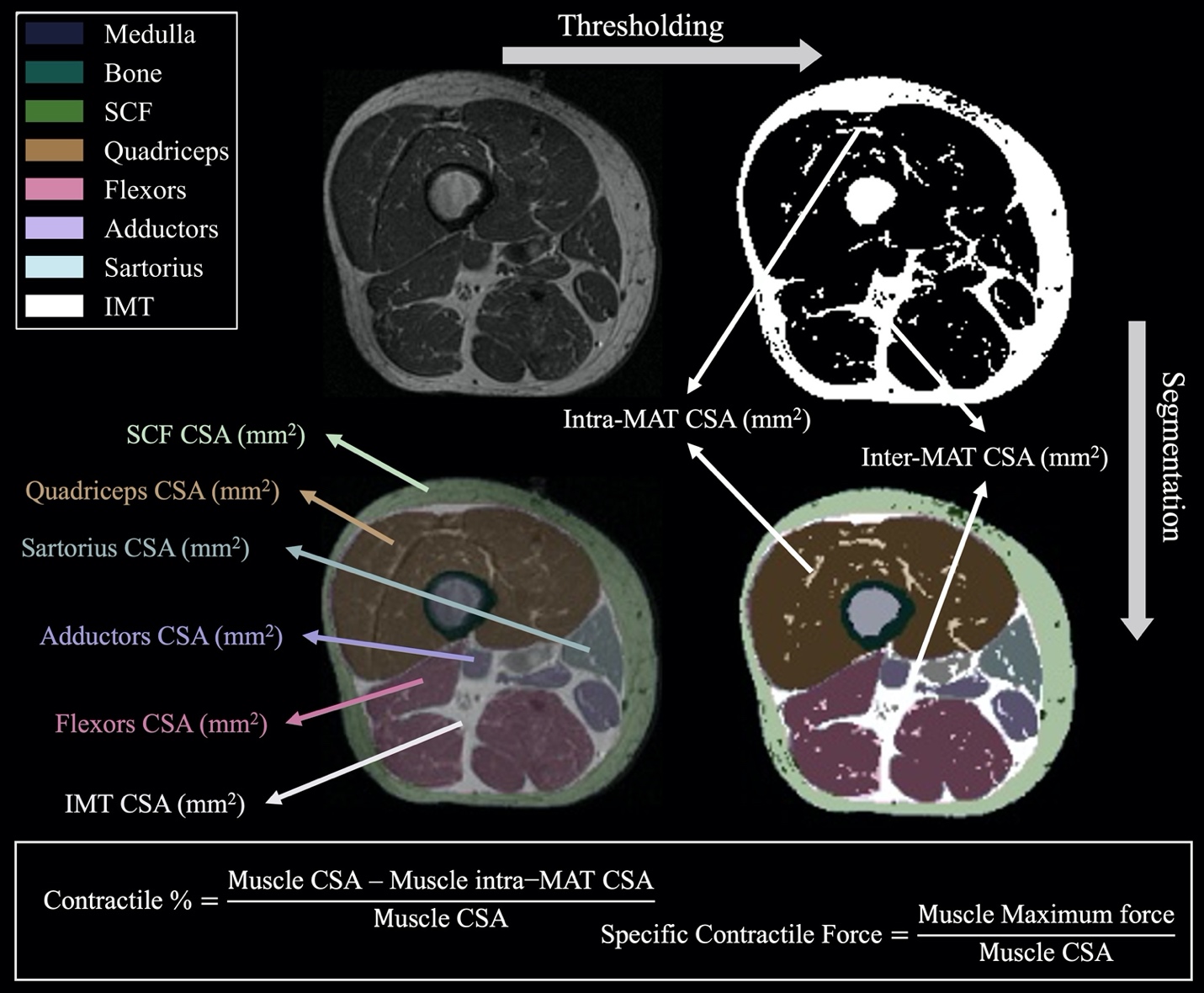


Illustration of study outcome variables. CSA: Cross-sectional Area, IMT: Inter-muscular Tissue, Inter-MAT: Inter-muscular Adipose Tissue, Intra-MAT: Intra-muscular Adipose Tissue, SCF: Subcutaneous Fat.

## Appendix References

1. Alberti KG, Zimmet P, Shaw J. Metabolic syndrome--a new worldwide definition. A consensus statement from the International Diabetes Federation. Diabet Med 2006;23(5):469-480. doi: 10.1111/j.1464-5491.2006.01858.x
2. Li C. Little's test of missing completely at random. Stata Journal 2013;13(4):795-809.
3. Resseguier N, Giorgi R, Paoletti X. Sensitivity analysis when data are missing not-at-random. Epidemiology 2011;22(2):282. doi: 10.1097/EDE.0b013e318209dec7
